# Restriction of russet mite and influence on predatory mite dispersal using different stem barriers in tomato crop

**DOI:** 10.1007/s10493-025-01064-0

**Published:** 2025-09-10

**Authors:** Elias Böckmann, Jule-Christine Spangenberg, Maximilian Dinkel

**Affiliations:** 1 Julius Kühn-Institut, Institute for Plant Protection in Horticulture and Urban Green, Messeweg 11/12, 38104 Braunschweig, Germany; 2 Hochschule Osnabrück, University of applied science, Albrechtstraße 30, 49076 Osnabrück, Germany

**Keywords:** Micula, Nano-Tape, Diatomaceous earth, Rapeseed oil, Physical barrier

## Abstract

The tomato russet mite, *Aculops lycopersici* (Tryon), is a key pest of commercially grown tomatoes worldwide. Due to its minute size, its detection is often not timely for effective control. In this study, the approach of limiting *A. lycopersici* population growth by repeated application of barriers on tomato stems is tested. In potted plant trials, barriers of oils, oil formulations, insect glue, diatomaceous earth [DE] and adhesive tape [NT] impeded upward movement of *A. lycopersici* on tomato stems for several weeks as compared to the control. Iolinid mites have shown promising results for *A. lycopersici* control. Therefore, the selectivity of barriers regarding predatory mites was also tested, using *A. swirskii* as a model organism. Barriers with [NT] showed the best selectivity ([NT] > oil formulation >[DE], *p* < 0.05 for each day count of mites above barriers). However, application of [NT] seems challenging in a broader scale. Under greenhouse conditions with practical crop stands, regularly applied barriers reduced the percentage of symptomatic leaves for two weeks (oil formulation and [DE], variety Roterno, 2023 and oil formulation, variety Baylee, 2024, *p* < 0.05; in 2024 no significant reduction was found on Roterno and [DE] was not tested). The comparison of different varieties showed less effect of barriers in the variety Roterno with more upward facing leaves, indicating that leave angle affects overcoming of barriers by *A. lycopersici*. The fact that under greenhouse conditions this control effect did not cover the whole season reduces the practical applicability of the method. Under similar conditions, an earlier study reached longer lasting control using insect glue as a barrier substance. Possible reasons for the discrepancy between studies include differences in tomato varieties, barrier positions and pest densities.

**Supplementary Information:**

The online version contains supplementary material available at 10.1007/s10493-025-01064-0.

## Introduction

The tomato russet mite, *Aculops lycopersici* (Tryon), is a key pest of commercially grown tomatoes worldwide, and more recently a notable increase was observed in central European greenhouse production (Duso et al. 2010; Vervaet et al. 2021, 2022; Pfaff and Böckmann 2023). Due to its minute size, its detection is often not timely (Pfaff et al. 2020; Vervaet et al. 2021, 2022) and is based on the developed plant symptoms, which can be misleading (Crüger et al. 2002). Symptoms of the feeding activity on the upper and lower epidermis of the tomato plant include the russet appearance of leaves and stem, in more severe stages even death of the plant (Royalty and Perring 1988; Capinera 2001; Duso et al. 2010). In our experience, the first symptoms tend to be the yellowing of the basal part of the leaflets, which are the ones closest to the stem. Effective control, especially based on selective acaricides, is limited, and consequently, research on effective natural enemies gains importance (Vervaet et al. 2021). Although several Phytoseiidae show promising capacity to feed and reproduce on a A. *lycopersici* diet, their impact on the pest on whole tomato plants is relatively low (van Houten et al. 2013). The reason is that the small-sized (150–200 μm) *A. lycopersici* are protected by the tomato trichomes from its bigger predators (Simmons and Gurr 2005; van Houten et al. 2013). Recently, smaller predatory mites such as *Homeopronematus anconai* (Baker) and *Pronematus ubiquitus* (McGregor) (Acari: Iolinidae) (225–280 μm) come into focus because they can prey on *A. lycopersici* between the trichomes (Pijnakker et al. 2020; Vervaet et al. 2022). If these promising predatory mites will provide full control is not yet clear. First studies under practical conditions show promising effects, but not always a full control (Pijnakker et al. 2020, 2022; Maret et al. 2024).

In this study, we examine another path to control *A. lycopersici*, taking advantage of its dispersal modes. For Eriophyoid mites, these modes include active dispersal, such as walking to uncolonized plant surfaces or neighboured plants in direct contact with the colonized plant, and passive dispersal, such as transferral via air currents or phoresy by using moving vectors as transport vehicle (Michalska et al. 2010). Phoresy is likely to occur by raindrop splashes, insects, or humans working in the crop (Jeppson et al. 1975; Capinera 2001). However, under greenhouse conditions in pure culture tomato crops where all neighboured plants are hosts, in relative comparison, active dispersal via walking is likely to be of the highest importance to reach new host plants (Pfaff et al. 2023). Because tomato plants are typically grown in a layer system, with plants reaching an enormous total length, reaching of uncolonized plant surfaces within one plant by active walking becomes a steady race of plant growth against colonization via active walking (Pfaff et al. 2023). A study of Pfaff et al. (2023) showed that plants can win this race if insect glue barriers are applied weekly on the plant stems, 15 cm below the plant tip. While this procedure effectively reduced damage symptoms, it is impractical to use insect glue in practice due to the nuisance of workers, soiling of materials and tedious application. To overcome those drawbacks, in the current study, more practicable materials were screened in single plant trials, and promising candidates were applied under greenhouse conditions. Furthermore, we investigate how the variety of tomato, and specifically the angle at which the leaves are positioned relative to the stem, influences efficacy of the method. We also consider the potential impact of the method on the application of predatory mites.

## Materials and methods

### Russet mite—potted plant trial

In all potted plant trials, depending on the replicate number, tomato plants of the variety Roterno (Roterno F1, organic, Rijk Zwaan, the Netherlands) were distributed on four or six tables, each table containing each treatment once or twice in separate randomized blocks. Distance between plants within blocks was at least 2 m. Plants were watered by hand and grown up to a height of about 40 cm in pots of 21 cm diameter. Then, leaves on the stem were cut off, leaving only two full-grown leaves on top of each plant. Plants were stabilized using a wooden stick connected only to the upper plant part, the stick was prepared with a 10 cm insect glue (Temmen Insektenleim, Germany) [IG] barrier to impede walking up of russet mite on this path. Then, barriers were, depending on the material, sprayed, spread or wrapped around the plant stem at a height of 15 cm in a width of 5 cm. The next day, about 1000 *A. lycopersici* of all stages from the rearing were transferred to the plants by leaning infested plant material against the stem base. *A. lycopersici* numbers for infestation were estimated by counting representable parts of the plant materials under the stereomicroscope, and total numbers were calculated by estimating the total area of the plant material. The same procedure was used for infestation in all experiments. Two days later, successful infestation was determined by visual control of the lower stem using a mobile digital microscope (Firefly, Belmont, USA). The number of *A. lycopersici* above the barriers was counted one day before infestation to ensure for a clean start and then weekly after infestation using tape imprints, a method described by (Pfaff et al. 2023). Additional plants without barriers were installed as control, and tape imprints were taken weekly at same height as for the treated plants. Mean temperature and relative humidity (r.h.) during experimental time were 21 °C, 41% r.h. at 12 am and 17 °C, 55% r.h. at 12 pm in 2023 and 22 °C, 46% r.h. at 12 am and 22 °C, 47% r.h. at 12 pm in 2024.

Barriers tested in 2023 included insect glue ring as positive control [IG], the rapeseed oil formulation Micula^®^ (Biofa, Germany) [M], diatomaceous earth spray (Ektosol Kieselgur Spray, Germany) [DE], rapeseed oil [RO], and Nano-Tape (Baurix, Germany) [NT], with four replicates per treatment and control [C]. Due to the promising results with [M] and [RO] in 2023, we focussed on plant-based oils and oil formulations in 2024 that are registered as plant protection products (PPP), basic substances or PPP-additives. Consequently, barriers tested in 2024 included [M], Trifolio S-forte (Trifolio-M GmbH, Germany) [TS] and sunflower-oil (Alnatura, France) [SO], with six replicates per treatment and control. [DE] and [M] are registered PPP, [SO] is registered as basic substance in Germany and [TS] is a PPP-additive.

### Russet mites—greenhouse trials

In 2023, a greenhouse trial was carried out adopting the approach used in (Pfaff et al. 2023). Seventy-two tomato plants of the variety Roterno were planted into three adjacent soil-greenhouses, each greenhouse containing 24 plants divided into six plots of four plants each (see also Pfaff et al. 2023). The minimum temperature of the greenhouses was set to 18 °C; whenever the temperature fell below this level, the heating system was activated. Roof windows were opened at temperatures above 22 °C, and side windows opened above 24 °C. There was no active cooling or humidity control in the greenhouses. The plants were grown in layer cultivation. Defoliation and harvest were conducted once per week, prior to the sampling of leaves and after winding tomatoes around the twines and removing of side shoots. Winding and shoot removal were done twice per week and separately from defoliation and harvest. Gloves and protective overalls were used and gloves were disinfected using EtOH 96% after every plot and both was changed after work was finished at a treatment. Each plant was infested on the 10th of May one day after the absence of *A. lycopersici* on plants was verified taking three sticky tape imprints per plant. For this purpose, infested tomato leaves and stems from the rearing, hosting an estimated number of 2000 *A. lycopersici* of all stages, were placed on a leaf at middle plant height. To test whether *A. lycopersici* did establish, all plants were checked for *A. lycopersici* individuals seven days post inoculation, with a mobile digital microscope. Physical barriers were applied as a ring around the stem of 5 cm width applied at upper (1.60 m height) and middle (1.20 m height) part of each plant after first symptoms became visible. After that, a new barrier was applied 40 cm above the highest barrier every 14 days. Starting with week 8, because symptoms increased in all treatments, the application was carried out every week and 20 cm above the last barrier, accounting for the expected growing length of the tomato plants in that time (Pfaff et al. 2023). Different treatments consisted of a control without application of barriers, and a barrier application of [M] or [DE] every second week and later every week. Each treatment was replicated 6 times and was organized in six randomized blocks containing one replicate per treatment within the same double row. The four plants per replicate were separated by a plant-free break of approximately 1 m from neighboured replicates. Two monitorings were carried out weekly, one day before the next application of barriers: (I) One tape imprint per plant was taken above the latest barrier to count *A. lycopersici* above the applied barriers and on a similar height at control plants. (II) The number of leaves with visible symptoms, as well as the full number of leaves per plant, were counted.

In 2024, as a reaction to the results from 2023, the trial was repeated and expanded. Two varieties of tomato were used, namely again Roterno and additionally Baylee (Baylee F1, organic, Enza Zaaden, The Netherlands). The varieties were chosen to check for differences in the angle at which the leaf protrudes between Baylee, the variety used by Pfaff et al. (2023), and Roterno, the variety used in the current study in 2023. Specifically it was expected that angles in Roterno are much smaller i.e. leave position is much more upwards. To measure this factor, shortly before infestation, four leave angles per plant (192 per variety in total) were measured by using a protractor that was positioned parallel in front of the stem and reading out the respective leave angle. Leaves were chosen randomly per plant at medium height. This year, four adjacent greenhouses were used, resulting in 48 plants per variety. Each variety was planted as a block of one double row per greenhouse, with breaks after every four plants, which were then used as one replicate. Treatments were the same for both varieties: without application of barriers [C], weekly application of [M] barriers and bi-weekly application of [M] barriers. Trials setup and application as well as monitoring procedures were the same as in 2023. Each treatment was replicated four times and was organized in four randomized blocks containing of one replicate per treatment and variety within the same greenhouse. Each plant was infested on the 30th of May.

### Predatory mites—laboratory trial

A laboratory trial was prepared using petri dishes. In each dish, a 3 cm piece of a tomato stem half (Roterno F1, Rjik Zwaan, Netherlands) with a semicircular diameter was placed centrally, embedded in a thin layer of [IG]. On each stem halve, a 1 cm barrier was applied, dividing the stem halve into a 0.3 cm part serving as starting area, and a 1.7 cm part serving as observation area. The next day, a single *A. swirskii* mite was released on the starting area, and the observation area was searched under the stereomicroscope every hour. *A. swirskii* was used as a model organism for other predatory mites that could be used in practice in the future, such as *P. ubiquitus*. No additional food was provided. If the mite was found in the observation area, this was counted as a success and the replicate was no longer observed. After 5 h, the experiment was stopped, and successful crossings over the barrier were summed up per treatment. The barriers contained of [M], [DE] and [NT], respectively. A [C] without barrier was prepared additionally. Per treatment and [C], 15 replicates were carried out.

### Predatory mites—potted plant trial

In a second step, the potted plant setup described earlier for russet mites was adapted to test for the effect of barriers towards predatory mites. Treatments were the same as tested before in the laboratory ([DE], [NT], [M], [C]), and barriers were applied one day before infestation. Using *A. swirskii* as a model organism, plants were prepared as described in the potted plant trials. Plants were infested with about 1000 *A. lycopersici* of all stages above and 2000 below the barrier in order to facilitate the movement of *A. swirskii* on the tomato stem by reducing trichome numbers (van Houten et al. 2013) and to attract them to the upper plant region. Infestation was carried out by attaching infested plant material to the stem base of the potted plants and by fixing the material with fine needles to the upper plant part. Application of *A. swirskii* was carried out 15 days later by distributing the content of a half sachet (Swirskii-Breeding-System, Biobest, Belgium) around the base of each plant stem. No additional food was provided. To estimate the number of *A. swirskii* per plant, 15 half sachets were frozen and sieved (mesh size 0.5 mm) into a petri dish, and predatory mites were counted under the stereomicroscope, resulting in 125 ± 22 (average ± standard deviation) mites per sample. Monitoring of *A. lycopersici* was carried out 2 cm above and below the barrier using the tape imprint method (Pfaff et al. 2020) to verify successful infestation. *A. swirskii* were counted using a handheld 10X magnifier and counting in an area of about 12 cm height around the stem, starting directly above the barrier. Both monitorings were carried out one day before infestation to ensure a clean start, and after infestation five times every three or four days. The trial was carried out with 10 replicates per treatment. Potted plants were distributed in a greenhouse on five tables, each table containing two randomized blocks. Mean temperature and humidity during experimental time was 20 °C, 68% at 12 am and 18 °C, 76% at 12 pm.

### Statistical analyses

#### Russet mites

In the potted plant trial, no statistic was applied because the barriers [RO], [IG], [M], [NT] and [TS] fully impeded *A. lycopersici* from reaching upper plant parts. Hence, the result is very clear, and the lack of variance with just zeros in treatments makes statistical analysis difficult.

In the greenhouse trial 2023, regarding numbers of *A. lycopersici* on tape imprints, a generalized linear mixed-effects model (GLMM) using the glmmTMB() function of the glmmTMB package (Brooks et al. 2017) was applied. Because of overdispersion, a negative-binomial dispersion (nbinom1) was assumed. The number of *A. lycopersici* above the last barrier was included as a response variable; treatment, rating date, their interaction, and a random intercept for the block were included in the model as explanatory factors. In the case of leaf symptoms caused by *A. lycopersici*, the same model was applied, now with the relation of symptomatic as compared to healthy leaves per plant included in the model using the cbind() function. As the first symptoms in 2023 were found in one block one week earlier than in the other two blocks, the application also started accordingly one week earlier here. For statistical analyses and figures, data of that block was shifted accordingly one week to align expected effects. In 2024, the same statistical approaches were taken, just the variety was included as an additional explanatory factor. Post-hoc tests with the emmeans package (Lenth 2021) were carried out considering only the effects within the same rating day (2023, 2024) and variety (2024).

#### Predatory mites

For the laboratory predatory mite experiment, we analyzed the proportion of *A. swirskii* that crossed the barrier (target area) as compared to those that remained in the starting area using a chi-squared test for independence followed by a pairwise comparisons of proportions with continuity correction using holm method for p-value adjustment.

In the potted plant trial, a GLMM using the glmer() function of the lme4 package (Bates et al. 2015) was performed assuming a Poisson distribution of data. The number of *A. swirskii* above the barrier was included as the response variable; treatment, rating date, their interaction, and a random intercept for the block were included in the model as explanatory factors. A post-hoc test with the emmeans package (Lenth 2021) was carried out considering only the effects within the same rating day.

All statistical analyses were carried out using R (R Core Team 2021).

## Results

### Russet mites

**Fig. 1:**
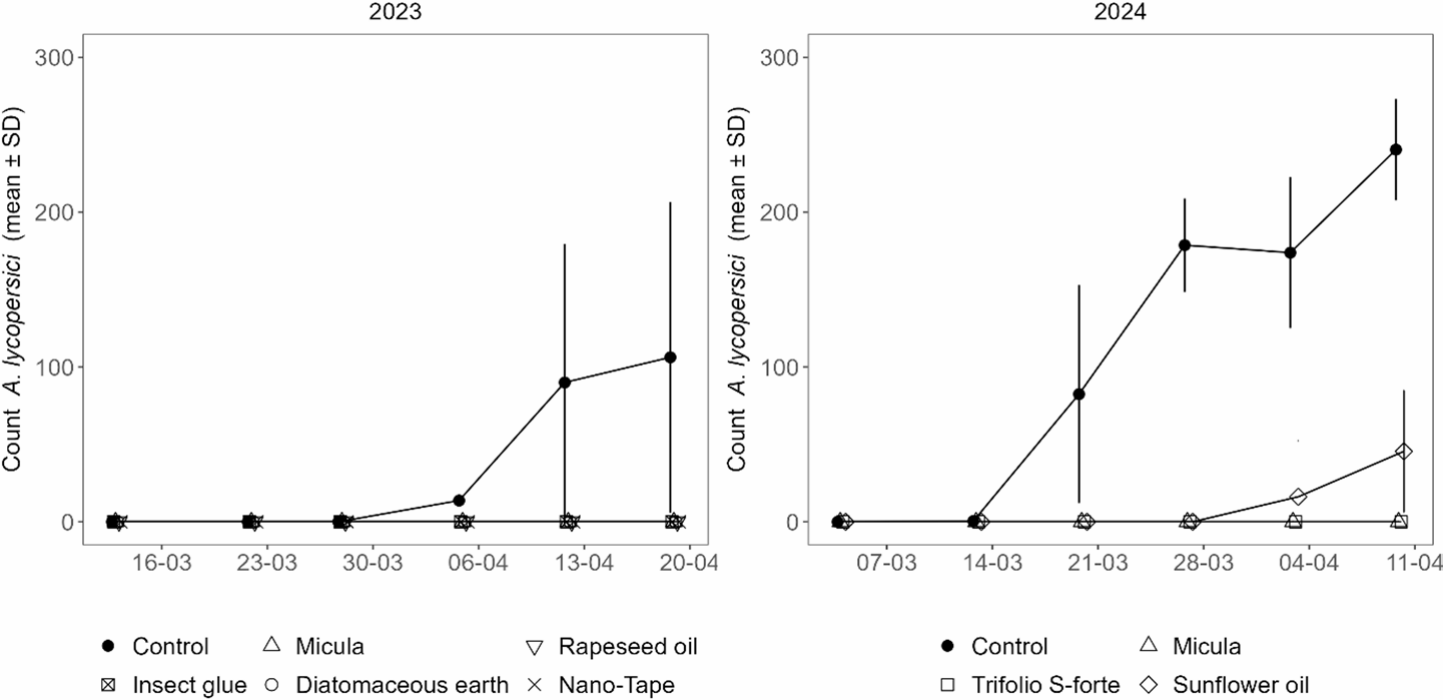
Counts of *A. lycopersici* above different barriers applied to stems of single potted tomato plants and on comparable plant stem height of the control without barrier. The russet mites were applied to the stem base on the 14th of March in 2023 and on the 5th of March in 2024. First count was carried out one day before application to ensure a clean start

The potted plant trials showed that several materials can impede the migration of *A. lycopersici* to upper plant parts. In 2023, all tested barriers consisting of [M], [DE], [NT], and [RO] as well as a positive control with [IG] delayed migration at least three weeks until the end of assessments as compared to [C] (Fig. 1).

**Fig. 2:**
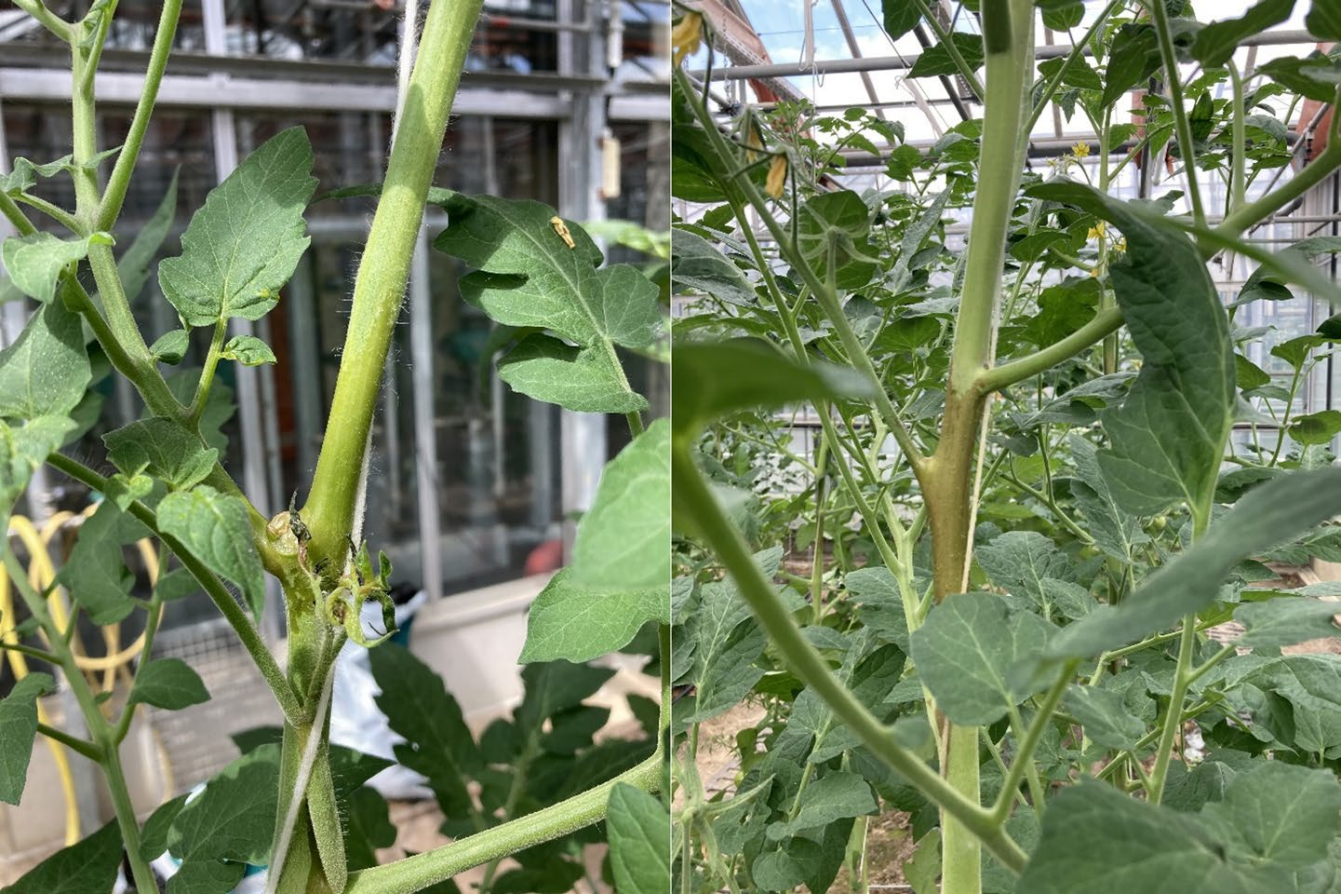
Phytotoxic symptoms on tomato stems some days after application of Micula^®^, including effect on a young side shoot (left) and after several weeks (right)

In 2024, where only oils and oil formulations were tested, barriers with [M] and [TS] delayed migration at least four weeks until the end of assessments, whereas [SO] delayed migration only two weeks as compared to [C] (Fig. 1). Phytotoxic effects on the stem were observed for [M], [SO] and [RO], whereas no symptoms were visible for [NT], [DE] or [IG]. Symptoms were a darker coloration locally at the application site for [M] and additionally black spots above and underneath that area for [SO] and [RO]. Plants did not show differences in growth or leaf condition as a result. Symptoms of oils and oil formulations at the application site were similar; exemplary symptoms from the greenhouse trial with [M] are shown in Fig. 2.

**Fig. 3:**
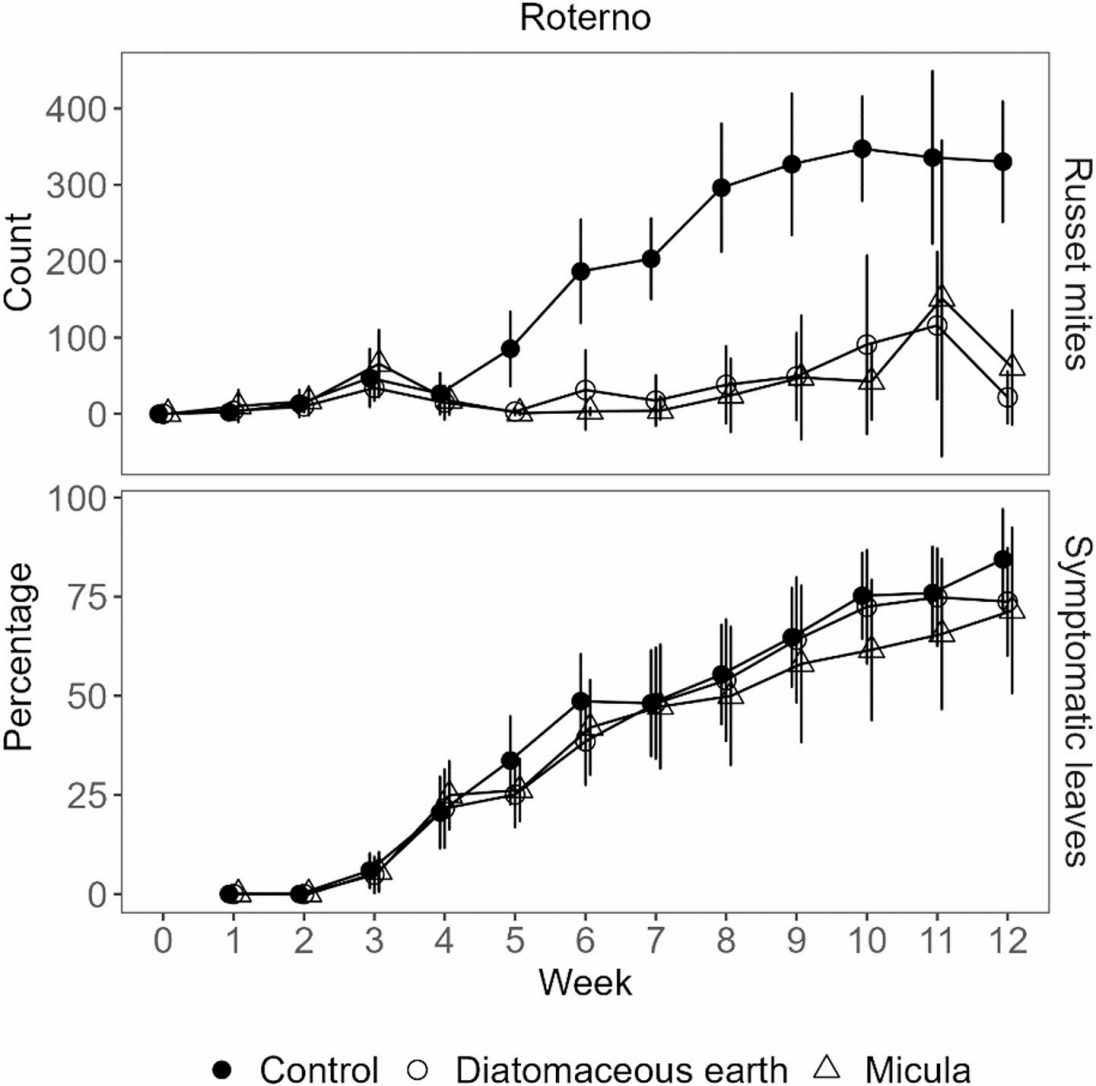
Russet mite counts on the tomato stem above barriers or at comparable height in the control (above) and percentage of symptomatic leaves (below) in small greenhouse tomato stocks in 2023. Graphs show mean values with error bars representing the standard deviation. Monitoring of russet mites started one day before inoculation with russet mites in week zero to ensure a clean start. Application started in week three after first symptoms were detected. Starting at week eight, application of barriers was switched from bi-weekly to weekly intervals

The application of [M] and [DE] in 2023 impeded migration to the stem directly above the last applied barrier as compared to the control tested at similar plant height (Fig. 3). Differences to the control were significant for [DE] already in week four (*p* < 0.05) and for both treatments from week five onwards until the end of the trial (*p* < 0.0001). There were no significant differences between [DE] and [M] treatments. Although numerically both treatments reduced leave symptoms similar in weeks five and six, effects were only significant for [DE] (*p* < 0.05). In weeks 10 and 11, [M] significantly reduced symptomatic leaves compared to the [C] and [DE] (*p* < 0.05) (Fig. 3).

**Fig. 4:**
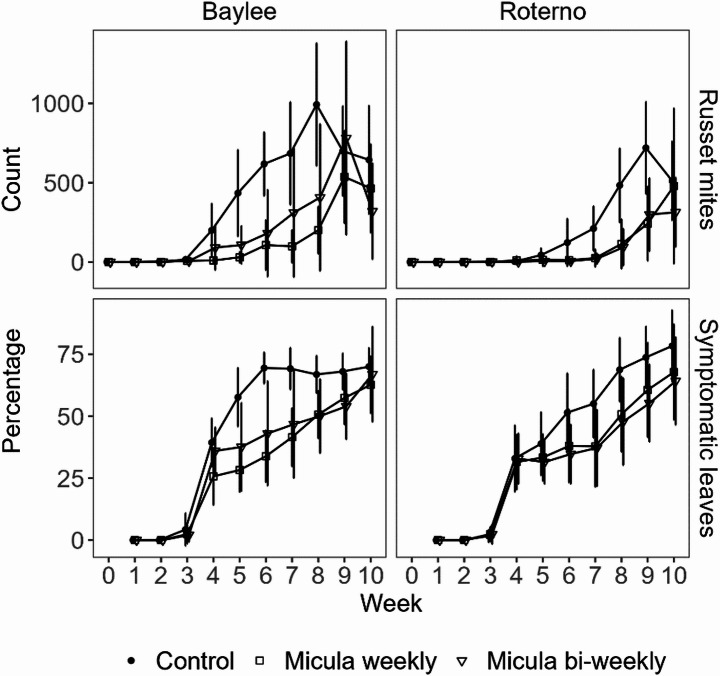
Russet mite counts on the tomato stem above barriers or at comparable height in the control (above), and the percentage of symptomatic leaves (below) in small greenhouse tomato stocks in 2024 for the varieties Baylee (right) and Roterno (left). Graphs show mean values with error bars representing the standard deviation. Monitoring of russet mites started one day before inoculation with russet mites in week zero to ensure a clean start. Application started in week three, after first symptoms were detected

In 2024, the tested varieties Baylee and Roterno showed a strong difference regarding the angles at which the leaves protrude, that is 99 ± 9 ° (mean ± SD) in Baylee and 52 ± 10 ° (mean ± SD) in Roterno. In the variety Baylee in 2024, only weekly application of [M] resulted in significantly reduced numbers of *A. lycopersici* above the barrier as compared to the [C] from week four to seven (weeks four and five: *p* < 0.0001, weeks six and seven: *p* < 0.05). Additionally, the difference between weekly as compared to bi-weekly application of [M] was significant in week four (*p* < 0.05). In the variety Roterno in 2024, weekly and bi-weekly application led to significant reductions compared to the [C] only in weeks six and seven (*p* < 0.05) (Fig. 4). The percentage of symptomatic leaves on the variety Baylee was significantly reduced in weeks five and six by weekly applications (*p* < 0.05), but not by bi-weekly applications, compared to the [C]. No significant differences were detected for the variety Roterno (Fig. 4).

Numerically, at dates with significant differences, the reduction in the percentage of symptomatic leaves detected in both study years between [C] and [M] with weekly applications was much higher in 2024 using Baylee (week five: 52%; week six: 51%) as compared to Roterno (week five: 15%; week six: 25%) (Fig. 4). The same is true for bi-weekly application in Baylee in 2024 (week five: 34%, week six: 38%) compared to Roterno in 2023 (week five: 24%; week six: 14%) (Figs. 3 and 4), although no direct comparison is possible here. In both years, phytotoxic effects were observed (Fig. 2). Effects stayed local and although not assessed quantitatively, no apparent effect on plant growth, leaf conditions, fruit production or -quality was observed apart from symptoms at the application site.

### Predatory mites

In the laboratory trial, 8, 5, 12 and 3 individuals of the model organism *A. swirskii* crossed the barrier and reached the target area in [C], [DE], [NT] and [M], respectively (Chi-squared test: df = 3, *p* = 0.006). The proportion of individuals reaching the target area was only significantly different between [NT] and [M] (*p* = 0.021).

**Fig. 5:**
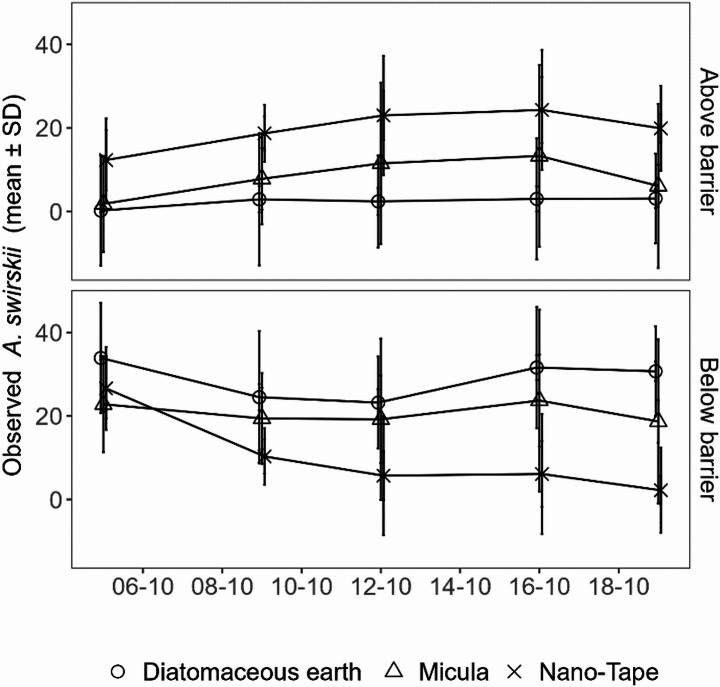
Counts of *A. swirskii* observed above and below different barriers applied to stems of single potted tomato plants. The predatory mites were applied to the stem base on October 4th

In the potted plant trial, observations of *A. swirskii* above the barrier showed strong differences from the first rating date and throughout rating dates, with the [NT] barrier being crossed by most *A. swirskii*, followed by [M]. Very few predatory mites were observed above the [DE] barrier (Fig. 5). Differences on each date were significant between all three treatments (rating dates one and five: *p* < 0.05, rating dates two, three and four: *p* < 0.0001). Trends observed below the barriers were just opposite to the ones above the barriers, descending to near zero *A. swirskii* in treatment [NT] 15 days after their introduction (Fig. 5). Tape imprints indicate that *A. lycopersici* remained established and followed similar trends in all replicates above and below the barrier from the first to the last assessment (supplementary Fig. 2). At the last assessment, a tape imprint below the barrier contained on average 18.6 [NT], 11.2 [DE] and 4.8 [DE] *A. lycopersici* (supplementary Table 1). In addition, total numbers of *A. swirskii* observed on plants per sampling date including individuals above and below the barrier remained very similar without clear trends (supplementary Fig. 2).

## Discussion

The aim of this study was to identify a practical and effective barrier substance that can be applied to the tomato crop to control *A. lycopersici* and that can be crossed by predatory mites.

Regarding the application of predatory mites, the best results in terms of selectivity and the absence of phytotoxic effects were obtained using [NT] barriers in both the laboratory and the potted plant trial. Furthermore, the low numbers of *A. lycopersici* found below the [NT] barrier at the final assessment date suggest that the [NT] barrier may have served as a trap and additionally as a barrier: It was observed that many *A. lycopersici* remain stuck on the tape’s surface at the end of the potted plant trial. However, there is no straightforward option for efficient application because the strips would have to be fixed manually to each stem, which is clearly too time-consuming for practice. Regarding [M] and [DE], no significant difference in selectivity, i.e. numbers of *A. swirskii* crossing barriers, was detected in the laboratory trial. However, in the potted plant trial, significantly more *A. swirskii* were able to cross the [M] barrier. Therefore, with regard to the application of other predatory mites to control *A. lycopersici* on tomato crop, [M] seems to be the better option for combined usage in pest control. However, *A. swirskii* was used here as a model organism to gain initial insights on whether predatory mites can overcome the tested barriers. Still, bevor suggesting a combined use of barriers and predatory mites, specific tests need to be carried out to clarify if these findings are also true for smaller iolinid mites, which are much more relevant in practice (Maret et al. 2024).

Furthermore, the application of [M] as compared to [DE] was more feasible under practical conditions. As tested in the greenhouse trial 2023, spray cans with [DE] often became clogged. However, when testing other [DE] formulations (data not shown), only the use of these ready-to-use spray cans showed to be effective. A drawback of [M] is the phytotoxic effect on the plant stem that was not detected for [DE]. However, the white colouring of [DE] would also complicate the marketing of hit fruits and impede photosynthesis of hit leaf surface. Although no precise yield assessment was carried out, the local phytotoxic effect of pure [M] barriers did not apparently reduce yield.

Pfaff et al. (2023) showed that [IG] barriers could effectively reduce *A. lycopersici* symptoms on tomato crop for nine weeks until the end of assessments compared to an untreated control. In the current study, the efficacy of the [M] barriers was 100% in the potted plant trial in 2023, preventing any *A. lycopersici* crossing. This was comparable to [IG], at least for the tested time period of three weeks in 2023, and [M] was still 100% effective after four weeks in the same trial setup in 2024 (Fig. 2).

Before that background, [M] and [DE] (2023) were selected for efficacy tests under practical conditions, as was [M] (2024). However, in 2023, neither [M] nor [DE] reduced symptoms on leaves markedly under greenhouse conditions. In 2024, the effects were also less consistent than in the study by Pfaff et al. (2023) using [IG]. Comparison of the methods used in both studies indicates some factors that may be the reason for these differences: One is that seemingly population density in the former study was much lower, as indicated by the much lower percentages of symptomatic leaves. While Pfaff et al. (2023) found in median only 20–40% symptomatic leaves in the control during the trial, percentages in the current study were much higher in both years. Mean values of 75% in 2023 for Roterno and of 70% and 78% for Baylee and Roterno in 2024, respectively, were detected at week 10 (Figs. 3 and 4). Also, the application of the stem barriers 15 cm below the tip, as done in Pfaff et al. (2023), may have helped to recapture the *A. lycopersici* population. In the current study application was done with a fixed distance to the latest barrier, which led to distances of up to approximately 1 m from the tip due to faster growth of the plants. Furthermore, in the current study in 2023, another variety, Roterno, was used compared to the former study. On Roterno, we found no or low numbers of *A. lycopersici* above the [M] barriers up to week 10 in 2023 and week 8 in 2024 (Figs. 3 and 4). In 2024, in the variety Baylee, higher numbers of *A. lycopersici* were found already at week 6 above the barriers of the weekly applications. Because leaves of Roterno protrude much more upright from the stem compared to Baylee, it is likely that these leaves of Roterno are colonized more quickly and formed bridges much more suitable for *A. lycopersici* to overcome stem barriers, as compared to the variety Baylee used in the former study. *A. lycopersici* apparently generally walk upwards, a behaviour described as non-compass negative gravitaxis (Grob et al. 2021). Indications for this behaviour were observed in the current study by accumulation of *A. lycopersici* at highest points on leaves and below barriers, similar as described before (Pfaff et al. 2023). Consequently, leaves that face more upwards can help to overcome stem barriers by serving as a direct bridge to upper leaves, or higher starting point for dispersal via airstream, as compared to more horizontally or downward protruding leaves. Results of the greenhouse trial in 2024 seem to support these assumptions, as there was only a significant reduction of symptomatic leaves in the variety Baylee but not in Roterno. In the same trial, the upward movement of the *A. lycopersici* population on the stem was pronounced faster on Baylee compared to Roterno, as visible in the stem counts from both varieties (Fig. 4). Still, control via stem barriers in the variety Baylee was more effective with regard to the reduction of leaf symptoms and *A. lycopersici* counts above stem barriers. Results generally indicate a faster population growth on the variety Baylee as compared to Roterno, which is in line with results of Christian Posch (LTZ Augustenberg, KA, Germany, personal communication). The results suggest that the stem plays a more significant role in the upward movement of *A. lycopersici* in varieties with horizontal or pendulous leaf orientation. This should be taken into account when applying barriers in practice.

Given the results of the potted trials in the current study, using [M] barriers could be a promising approach to control *A. lycopersici* in practice. However, the current study was unable to show the expected effects in greenhouse trials under practical conditions. Reasons for this could be the high densities of *A. lycopersici* in general and that these densities on control plants triggered regular infestation of treated plants above applied barriers. To confirm whether the efficacy of [M] barriers is comparable to that of [IG] and suitable for the continuous control of *A. lycopersici* under practical greenhouse conditions, it is necessary to treat full crop stands in greenhouses to prevent migration from highly infested adjacent control plants. Such trials are highly important to proof if practicable stem barrier methods are suitable to control *A. lycopersici* under real world conditions. Still, given the observed phytotoxic effects, a very accurate local treatment of plants and a registration of pure [M] for this indication would be needed. In this regard, the assessment of yield effects should be included as well. The efficacy of barriers with diluted [M] should additionally be tested to reduce the phytotoxic effects.

## Supplementary Information

Below is the link to the electronic supplementary material.

Supplementary Material 1 (81 KB)

## Data Availability

The datasets generated during and analyzed during the current study are available from the corresponding author on reasonable request.
