# Supplementary material for: Restriction of russet mite and influence on predatory mite dispersal using different stem barriers in tomato crop
Source: Exp Appl Acarol. 2025 Sep 10;95(3):39. doi: 10.1007/s10493-025-01064-0 (PMC12423254; doi:10.1007/s10493-025-01064-0)

Supplementary table 1

Mean numbers and standard deviation (SD) of *A. lycopersici* on sticky tape imprints taken on the first and last assessment of the potted plant – predatory mite trial, taken above and below the respective barrier.

| Barrier | Position to barrier | First Assessment (mean ± SD) | Last Assessment (mean ± SD) |
| --- | --- | --- | --- |
| Diatomaceous earth | above | 59.3 ± 31.6 | 30.6 ± 22.5 |
| Diatomaceous earth | below | 78.5 ± 45.0 | 18.6 ± 18.9 |
| Micula | above | 32.9 ± 42.8 | 18.6 ± 23.2 |
| Micula | below | 41.1 ± 53.1 | 11.2 ± 14.7 |
| Nano-Tape | above | 44.2 ± 35.3 | 18.5 ± 8.9 |
| Nano-Tape | below | 94.5 ± 38.9 | 4.8 ± 4.5 |

Supplementary figure 2

Counts of *A. swirskii* (AS) observed in total as well as AS and *A. lycopersici* (AL) observed above and below different barriers applied to stems of single potted tomato plants.


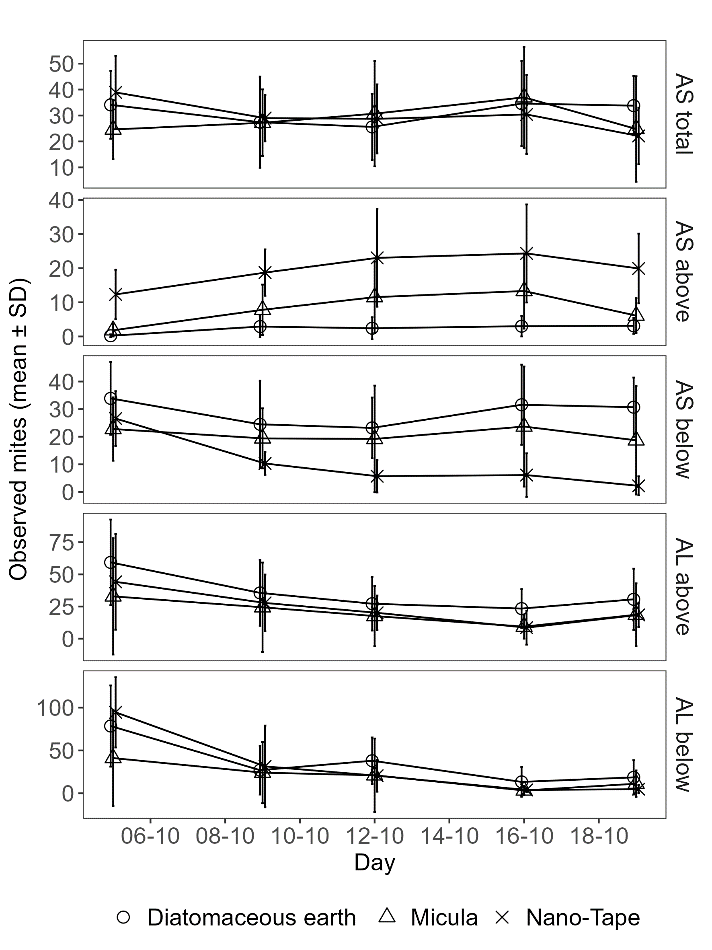

Supplement: Supplementary file 1 — Supplementary Material 1 (81 KB) [file 10493_2025_1064_MOESM1_ESM.docx]
